# PUP-IT2 as an alternative strategy for PUP-IT proximity labeling

**DOI:** 10.3389/fmolb.2022.1007720

**Published:** 2022-09-29

**Authors:** Suyu Yue, Peng Xu, Zhihe Cao, Min Zhuang

**Affiliations:** School of Life Science and Technology, ShanghaiTech University, Shanghai, China

**Keywords:** PUP-IT, ligase, protein labeling, proximity tagging, PafA

## Abstract

PUP-IT is a proximity labeling method based on the prokaryotic enzyme PafA. PafA mediates the ligation of Pup, a small peptide, to the proximal proteins. It is different from other proximity labeling methods, such as BioID and APEX, in that both the enzyme and the labeling tag are proteins, which allows for potential *in vivo* applications. All proximity labeling involves the genetic fusion of the proximity labeling enzyme with the bait protein. However, PafA is a 55 kDa enzyme which sometimes interferes with the bait function. In this study, we tested an alternative proximity labeling strategy, PUP-IT2, in which only a small 7 kDa protein is fused to the bait protein. We examined the activity of PUP-IT2 *in vitro* and in cells. We also compared it with the original PUP-IT. Finally, we applied PUP-IT2 coupled mass spectrometry to map protein-protein interactions. Overall, we established a new way to use PUP-IT2 for proximity labeling, and this method may have a broad application.

## Introduction

Proximity labeling is a method where the proximity labeling enzyme is genetically fused to the protein of interest (POI), and the substrate of the enzyme is catalyzed to be attached to the proximal proteins of POI. Different proximity labeling systems, including BioID (Roux et al., 2012), NEDDylator (Zhuang et al., 2013; Hill et al., 2016), APEX (Martell et al., 2012), and PUP-IT (Liu et al., 2018) have been generated based on different types of proximity labeling enzymes. BioID adapts a mutated biotin ligase BirA, NEDDylator utilizes a NEDD8 conjugating enzyme, APEX fuses a peroxidase, and PUP-IT applies a Pup ligase PafA.

Over the past few years, proximity labeling has emerged as a powerful approach and has already been applied to study a wide range of biological processes (Paek et al., 2017; Phelan et al., 2018; Qin et al., 2021). Proximity labeling has mainly been used to map local protein interactomes (Coyaud et al., 2015; Lobingier et al., 2017; Paek et al., 2017) and map protein-DNA (Myers et al., 2018; Qiu et al., 2019) or protein-RNA interactions (Kaewsapsak et al., 2017; Padron et al., 2019; Zhang et al., 2020). Given the importance of proximity labeling, continuous efforts have been made to improve the proximity ligation by increasing the activity of the enzyme *via* protein engineering (Lam et al., 2015; Branon et al., 2018), reducing the labeling background with split-enzyme strategies (De Munter et al., 2017; Han et al., 2019; Cho et al., 2020), or simply reducing the size of the enzyme by homologous replacement (Kim et al., 2016). Using smaller enzymes tends to minimize the functional interference with the tagged POI.

PUP-IT is a proximity tagging system developed recently. In prokaryotic cells, prokaryotic ubiquitin-like protein (Pup) is covalently attached to a target protein by the ligase PafA, tagging substrates for bacterial proteasomal degradation. PUP-IT system involves the expression of POI-fused PafA and its substrate Pup in the active form as PupE with the C terminus Gln mutated to Glu. PafA utilizes ATP to generate a C terminal γ-glutamylphosphate on PupE, which is poised for the nucleophilic attack by a substrate lysine side chain to form the covalent bond. Unlike other proximity labeling system, which depends on the diffusion of activated substrates, PUP-IT keeps the activated PupE bound to the enzyme, thus having a limited labeling radius. PUP-IT has been used to discover physical contact sites between peroxisomes and mitochondria (Huo et al., 2022), to map RNA-protein interactions (Zhang et al., 2020), and to identify new protein-protein interactions (Wang et al., 2019; Zhang et al., 2022; Zheng et al., 2022). Furthermore, other method, such as PUPIL (Xie et al., 2021), has been developed based on PUP-IT.

Despite a handful of applications of PUP-IT, PafA has the largest molecular weight among all the proximity labeling enzymes. The 55 kDa PafA might interfere with the structure and function of some fused POIs. To extend the application of PUP-IT, we design and test an alternative proximity labeling strategy, PUP-IT2, in this study. We show that proximity labeling mediated by PUP-IT2 occurs both *in vitro* and in cells. The PUP-IT2 allows minimal fusion to the POI, which potentially improves the application of the PUP-IT proximity labeling when the enzyme size matters.

## Results

### The design and validation of PUP-IT2 *in vitro*

**FIGURE 1:**
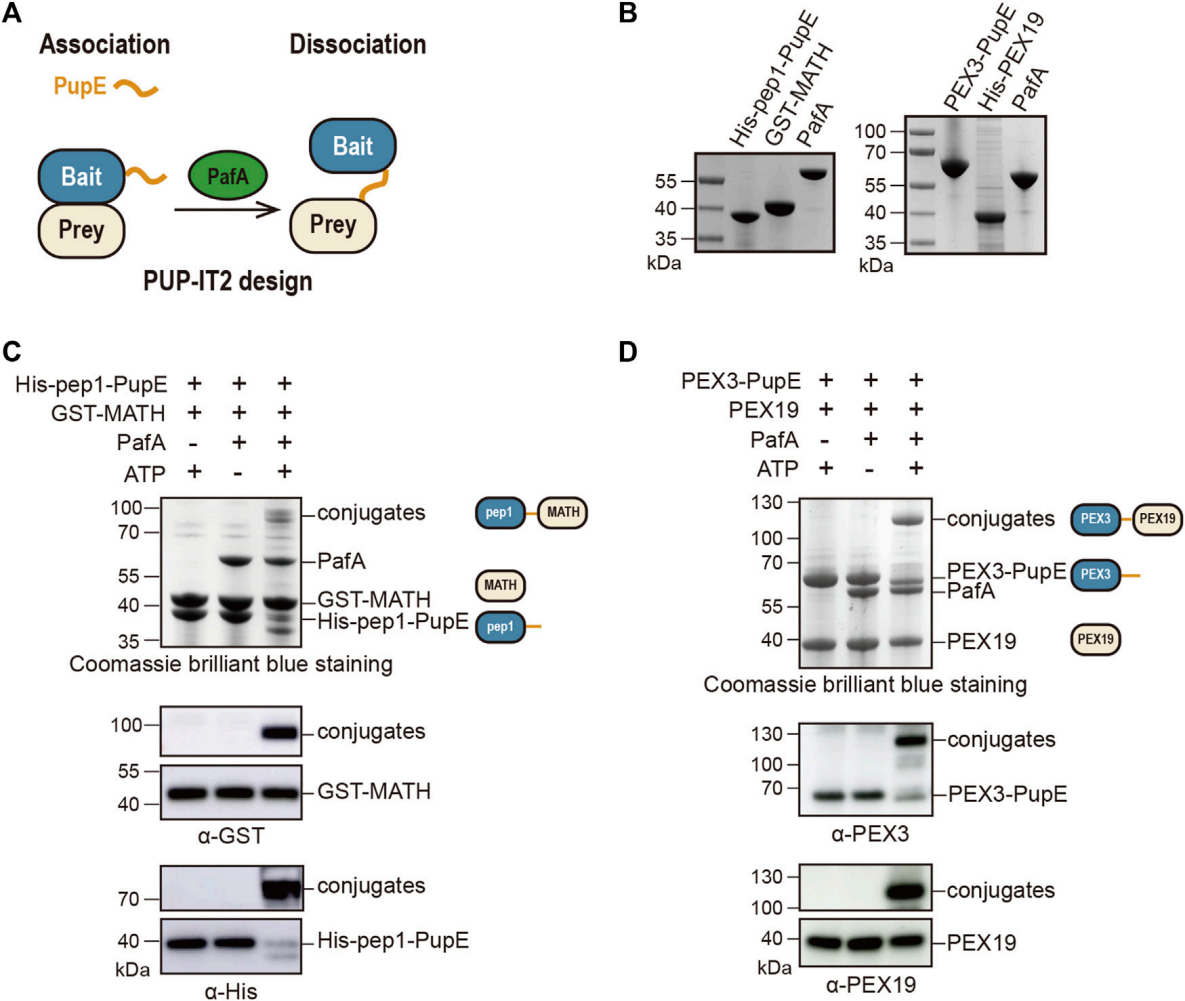
Design and validation of the PUP-IT2 proximity-tagging system. **(A)** Schematic of the PUP-IT2 design. BCCP-PupE (orange) is fused to a bait protein (blue), PafA (green) mediates the covalent attachment of PupE to prey proteins (yellow). **(B)** Coomassie brilliant blue staining of recombinant proteins His-SUMO-BCCP-pep1-PupE (labeled as His-pep1-PupE), GST-MATH, PafA, His-SUMO-Myc-PEX3-PupE (labeled as PEX3-PupE) and His-PEX19. 6 µg proteins were loaded on each lane. **(C)** *In vitro* Pup modification assay of PUP-IT2^pep1^. Using recombinant proteins purified as shown in **(B)** to set up the *in vitro* reaction at 37°C for 30 min 10 µM His-pep1-PupE and 10 µM GST-MATH were used for the reaction. Proteins are analyzed with SDS-PAGE for Coomassie stain and western blotting, using anti-His and anti-GST antibodies. Schematics are shown next to the protein bands with the same color code in Figure 1A to indicate the bait and prey proteins in the reaction system. **(D)** *In vitro* Pup modification assay of PUP-IT2^PEX3^. Using recombinant proteins purified as shown in **(B)** to set up the *in vitro* reaction at 37°C for 30 min 10 µM PEX3-PupE and 10 µM His-PEX19 were used for the reaction. Proteins were analyzed with SDS-PAGE for Coomassie stain and western blotting, using anti-PEX3 and anti-PEX19 antibodies.

To design the PUP-IT2, we fused PupE to the POI (bait) and expressed the free PafA in the system. When bait protein is in proximity with the prey protein, PafA will mediate the ATP-dependent activation of PupE C-terminal glutamic acid and the further ligation of PupE C terminus to the side chain of a lysine on the prey protein. Thus, the bait and prey proteins will be linked covalently *via* the PupE protein (Figure 1A). Further protein enrichment and characterization can be carried out using the affinity tag fused on PupE.

We first validated this design using two model protein-protein interaction systems. In a previous study, we used a pair of well-characterized interacting proteins, including the MATH domain of SPOP protein and the MATH-interacting peptide pep1, to examine the labeling efficiency of PUP-IT (Liu et al., 2018). The known dissociation constant between MATH and pep1 is 3.7 μM (Zhuang et al., 2009), which is considered as weak interactions. With PUP-IT2, we used the same system. GST was fused to the N terminus of SPOP MATH domain (GST-MATH), which has a molecular weight of 41 kDa in total, while pep1 was fused to the N terminus of PupE (His-pep1-PupE). The N terminus of pep1 was also fused with the BCCP domain for biotin labeling and SUMO for recombinant expression with a combined molecular weight at 30 kDa. The expression and purification of all proteins for PUP-IT2 labeling have been validated (Figure 1B). When GST-MATH and His-pep1-PupE were mixed in the presence of PafA, the interaction between MATH and pep1 would bring PupE to GST-MATH, where PafA mediates the formation of a covalently linked complex between His-pep1-PupE and GST-MATH with expected molecular weight at 71 kDa. Indeed, in the *in vitro* pupylation assay when both ATP and PafA were supplemented, a stable complex with larger molecular weight was detected in the denatured condition with Coomassie staining. Immunoblots further confirmed the presence of both MATH and pep1 in the gel band between 70 and 100 kDa (Figure 1C). We also tested two other MATH binders, pep2 (K_d_ = 76 μM) and pep3 (K_d_ = 266 μM) with the PUP-IT2 system. No obvious modification can be observed with these two weak binders.

We tested PUP-IT2 in another protein-protein interacting system, where PEX3 is the bait and PEX19 is the prey. PEX3 and PEX19 are both required for peroxisome biogenesis. PEX3 is a transmembrane protein with the N terminus forming a single transmembrane helix (residues 1–48) to anchor on the peroxisomal membrane. The C-terminal domain of PEX3 binds cytosolic PEX19 with a dissociation constant at 10 nM (Schmidt et al., 2010). We removed the transmembrane helix from PEX3 and fused PEX3 (residues 49–373) to PupE. PEX19 was fused to His tag to facilitate protein purification. Both protein fusions were expressed in *E. coli* and purified, as shown in Figure 1B. Similar to the MATH-pep1 system, the interaction between PEX3 and PEX19 is sufficient to mediate the formation of a covalent complex between these two fusion proteins in the presence of ATP and PafA (Figure 1D).

### Protein-protein interaction enhances PUP-IT2 labeling

**FIGURE 2:**
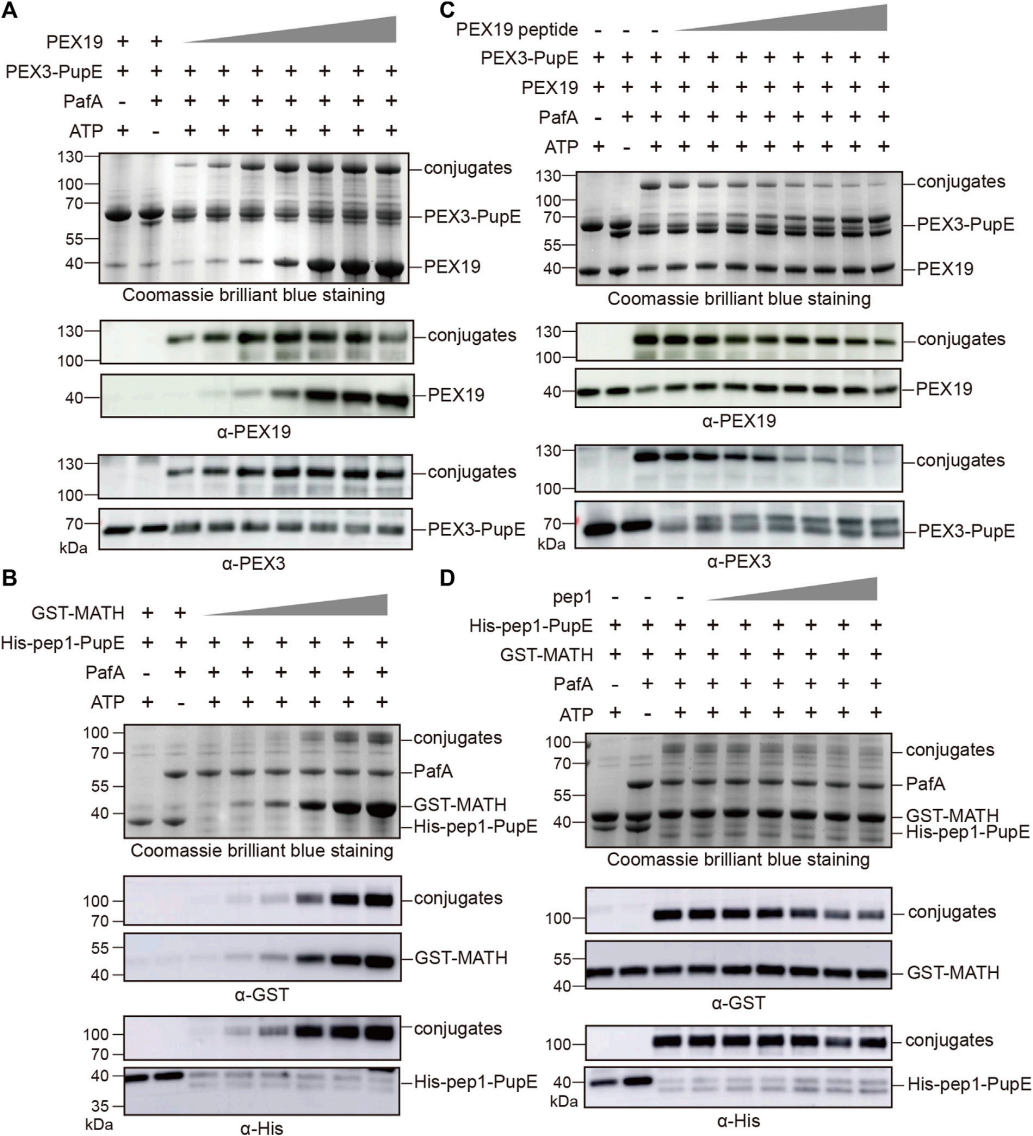
PUP-IT2 system labels specific interacting proteins. Coomassie stain and western blotting to examine the formation of protein conjugates. **(A)** In the PUP-IT2^PEX3^ *in vitro* reaction system, PEX19 was added at the concentrations from 1.25 µM to 30 µM (1.25, 2.5, 5, 10, 20, 25, and 30 µM). **(B)** In the PUP-IT2^pep1^ *in vitro* reaction system, GST-MATH was added at the concentrations from 1.25 µM to 40 µM (1.25, 2.5, 5, 10, 20, and 40 µM). **(C)** Free PEX19 peptide was dosed in to the PUP-IT2^PEX3^ *in vitro* reactions containing 10 µM PEX3-PupE, and PEX19 peptide concentrations varied from 20 μM to 2.5 mM (20, 40, 80, 160, 320, 640, 1300, 2500 µM). **(D)** Free pep1 peptide was dosed in to the PUP-IT2^pep1^ *in vitro* reactions containing 10 µM His-pep1-PupE, and pep1 peptide concentrations varied from 20 μM to 0.48 mM (20, 40, 80, 160, 320, and 480 µM).

To further confirm the specificity of the PUP-IT2 labeling system, we performed two other types of experiments. First, in the *in vitro* pupylation assay, we tested the labeling efficiency for PUP-IT2 with different substrate concentrations. With fixed PEX3-PupE concentration at 10 μM, PEX19 was added to the reaction with a series of concentrations ranging from 2.5 μM to 20 μM (Figure 2A). Even at the lowest PEX19 concentration, the conjugation could be detected with Coomassie staining. Western blots further confirmed the formation of the protein conjugates. The level of PEX3-PEX19 conjugates also increased with more PEX19. Similarly, the MATH/pep1 system was examined for labeling efficiency at low GST-MATH concentrations (Figure 2B). Despite the low affinity between MATH and pep1, the conjugates could be detected by western blots with 2.5 μM GST-MATH.

Secondly, a competition assay was used to inhibit specific PUP-IT2 labeling. It was known that the N terminal region (residues 14–33) on PEX19 is responsible for PEX3 interaction (Schmidt et al., 2010). Therefore, a synthetic peptide containing PEX19 (residues 14–33) would inhibit PEX3/PEX19 interaction, thus, inhibiting PUP-IT2 labeling. PEX19 (residues 14–33) was synthesized and named as PEX19 peptide. In the *in vitro* pupylation assay, the formation of PEX3-PEX19 conjugates was significantly reduced in the presence of PEX19 peptide, while PEX3 formed larger molecular weight conjugates with PEX19 peptide (Figure 2C). Similar experiments were performed with the MATH/pep1 PUP-IT2 system. Untagged pep1 was synthesized and added to the reaction to compete with His-pep1-PupE for binding to the MATH domain. With increasing free pep1 concentration, less MATH was conjugated with His-pep1-PupE (Figure 2D).

Moreover, to further exclude the potential non-specific labeling of PUP-IT2 with the *in vitro* experimental settings, we used bovine serum albumin (BSA) as an internal control to examine non-specific labeling. When BSA was added to the reaction system, the protein conjugates only formed between interacting protein pairs but not with BSA (Supplementary Figure S1). Taken together, these results suggest that the PUP-IT2 labeling system is well suited for mediating the covalent linkages between interreacting proteins.

### Validation of PUP-IT2 in cells

**FIGURE 3:**
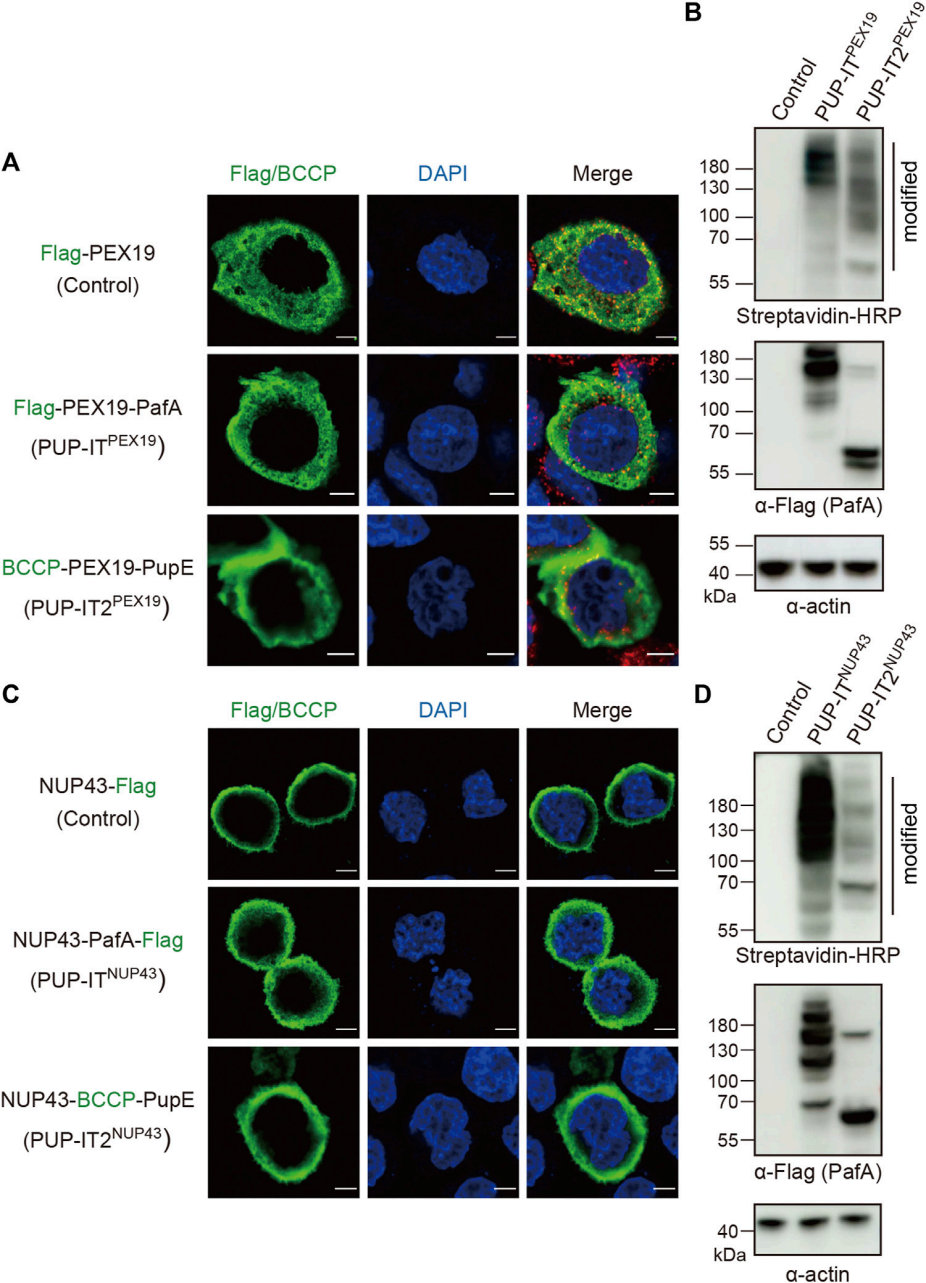
PUP-IT2 mediates proximity labeling in cells. **(A)** Immunofluorescence staining of HeLa cells transfected with Flag-PEX19, PUP-IT^PEX19^ (Flag-PEX19-PafA) or PUP-IT2^PEX19^ (BCCP-PEX19-PupE). Cells were stained with anti-Flag or cy3-conjugated streptavidin (green), and the nucleus were stained with DAPI (blue). Scale bars, 5 μm. **(B)** Immunoblots of ligated proteins in HEK293 cells transfected with empty vectors (control), PUP-IT^PEX19^ (Flag-PEX19-PafA, BCCP-PupE) or PUP-IT2^PEX19^ (BCCP-PEX19, Flag-PafA). HRP-conjugated streptavidin and other indicated antibodies were used for western blots. **(C)** Immunofluorescence staining of HeLa cells transfected with NUP43-Flag, PUP-IT^NUP43^ (NUP43-PafA-Flag) or PUP-IT2^NUP43^ (NUP43-BCCP-PupE). Cells were stained with anti-Flag/cy3-conjugated streptavidin (green) antibodies, and the nucleus were stained with DAPI (blue). Scale bars, 5 μm. **(D)** Immunoblots of ligated proteins in HEK293 cells transfected with empty vectors (control), PUP-IT^NUP43^ (NUP43-PafA-Flag, BCCP-PupE) or PUP-IT2^NUP43^ (NUP43-BCCP-PupE, Flag-PafA). HRP-conjugated streptavidin and other indicated antibodies were used for western blots.

Next, we addressed whether PUP-IT2 mediates proximity labeling in cells. The original PUP-IT design was used as a control for comparison. PUP-IT^PEX19^ was generated by fusing the Flag-tagged PafA to the C-terminus of PEX19, and PUP-IT2^PEX19^ by fusing BCCP-tagged PupE to the C-terminus of PEX19. HeLa cells were transfected with either PUP-IT or PUT-IT2 plasmids. The localization of each PEX19 fusion protein was examined *via* immunofluorescence (IF) staining with either Flag (for PUP-IT) or biotin (for PUP-IT2). The fusion of PafA or PupE did not affect the cytosolic distribution of PEX19 as expected (Figure 3A). The enzymatic activity of proximity labeling was evaluated *via* immunoblots (IB). PUP-IT^PEX19^ was co-transfected with BCCP-PupE, while PUP-IT2^PEX19^ was co-transfected with Flag-PafA in HEK293 cells. The expression of the PafA enzyme in either fused or unfused form was detected by anti-Flag IB. The activity of the proximity labeling was indicated by streptavidin-HRP blots. Although the PafA enzyme was expressed at a lower level in the PUP-IT2 system, the overall labeling for PUP-IT2 was as robust as PUP-IT (Figure 3B), reflecting the efficient PUP-IT2 ligation in cells.

We also used another protein, NUP43, a nuclear pore complex (NPC) component, to analyze the labeling efficiency of the two systems on membranes. Correct localization of NUP43-PafA-Flag (PUP-IT) and NUP43-BCCP-PupE (PUP-IT2) was confirmed by fluorescence microscopy, showing predominantly nuclear membrane localization (Figure 3C). Immunoblot of biotin-containing proteins suggest both systems mediate BCCP-PupE conjugations in cells (Figure 3D). Although the PafA enzyme was expressed relatively high in the PUP-IT2^NUP43^ transfected cells, less PafA self-modification occurred (Figure 3D), suggesting potential advantages of PUP-IT2 with less unwanted self-modification.

### PUP-IT2 is as efficient as PUP-IT for cellular labeling

**FIGURE 4:**
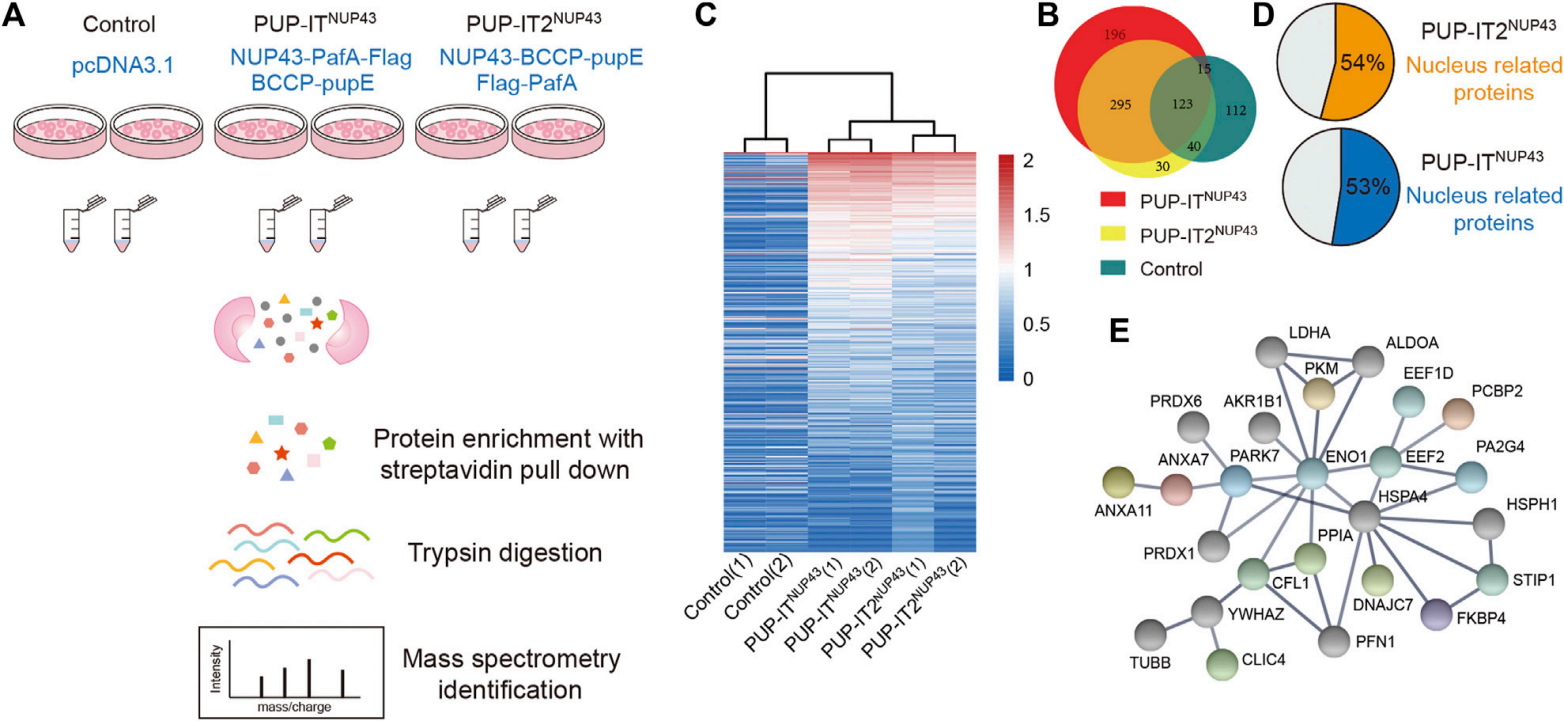
PUP-IT2 is as efficient as PUP-IT for cellular labeling. **(A)** The workflow of PUP-IT2 based proximity labeling to identify interacting proteins. **(B)** The proteins identified by PUP-IT^NUP43^ or PUP-IT2^NUP43^ proximity labeling. The relative abundance of proteins identified by PUP-IT^NUP43^, PUP-IT2^NUP43^ or control is depicted in the Venn diagram. **(C)** Proteins identified by PUP-IT^NUP43^, PUP-IT2^NUP43^ or control is depicted in the Heatmap diagram based on mass spectral counts. The color scheme represents the normalized MS count changes in log scale. **(D)** A pie chart showing the percentage of nucleus-associated proteins. The subcellular localization of proteins with a MS count 9 times higher than that of the control group were used for SRING gene ontology (GO) analysis. **(E)** STRING protein association network analysis of the nucleus-related proteins identified with PUP-IT2^NUP43^. The colored dots represent proteins, for which nuclear localization is suggested by Uniprot.

Previously, NUP43 has been used as a model system to compare the proximity labeling efficiency of BioID and BioID2 (Kim et al., 2016). In order to compare PUP-IT and PUP-IT2 proximity labeling properties in cells, we also adopted this system. We scaled up HEK293 co-transfection with different PUP-IT plasmid sets. Cells transfected with empty vector pcDNA3.1 were used as a control. The biotin-containing BCCP tag on PupE allows us to carry out the isolation of PupE conjugates under denaturing conditions, eliminating the presence of proteins that are associated with the target protein in a non-covalent manner. Following the experimental procedure in Figure 4A, we isolated PupE ligated proteins using streptavidin-coated beads and subjected these affinity-enriched proteins to proteomic analyses by mass spectrometry. With two biological experimental repeats, proteins identified with at least two unique peptides were used for analysis. In total, 488 proteins were identified in PUP-IT2^NUP43^, and 629 proteins were identified in PUP-IT ^NUP43^. Of note, 295 proteins were commonly detected by PUP-IT and PUP-IT2, representing a large overlap of labeling proteins between different methods (Figure 4B). 30 proteins were uniquely identified in the PUP-IT2 samples, whereas 196 proteins were only identified in the PUP-IT samples (Figure 4B, Supplementary Table S1). Both PUP-IT systems validated several previously described direct interactors of NUP43, including TCP1, NUDC, and KIF5B, which were selectively enriched in PUP-IT/PUP-IT2 samples compared to the controls (Supplementary Table S1).

To further compare PUP-IT and PUP-IT2, the spectral counts were analyzed in a heat map to highlight the enriched proteins in each sample (Figure 4C). The experiments have good reproducibility, with the two biological experimental repeats showing similar protein enrichment patterns. 152 and 83 candidate proteins were found to be enriched more than nine-fold in PUP-IT^NUP43^ and PUP-IT2^NUP43^, respectively, when compared with the control samples (Supplementary Table S2). Among these enriched proteins, more than half were nucleus proteins, consistent with the role of NUP43 as one subunit of the nuclear pore complex (Figure 4D). Further protein-protein interaction analysis revealed an interaction network for those proteins enriched in PUP-IT2 (Figure 4E), suggesting the proximity labeling occurred close to the nucleus.

Overall, when proximity labeling is coupled with mass spectrometry identification, PUP-IT2 is as efficient as PUP-IT for potential interacting protein identifications.

## Discussion

To minimize the size of the fusion protein, previous studies have been focusing on engineering the proximity ligation enzymes. PUP-IT is different from other proximity labeling methods in the way that both the enzyme and enzyme substrate are proteins, which allows the genetic fusion of either one.

In this study, we have developed a unique alternative proximity labeling strategy, PUP-IT2, in which PupE, a 64 amino acid protein (7 kDa), is fused to the POI. Compared to PafA (55 kDa) fused PUP-IT, PUP-IT2 has a fusion strategy with a significantly smaller fusion protein. Functionally, PUP-IT2 is comparable to PUP-IT in the ability to label proximal proteins *in vitro* and in cells. In addition, PUP-IT2 may provide other advantages. In Figure 3D where the anti-Flag antibody was used to probe NUP43-PafA protein level in cells, multiple high-molecular-weight bands occurred due to the in-cis modification of NUP43 by the fused PafA. By contrast, PafA was expressed as an untagged enzyme in the PUP-IT2 system, which is unlikely to self-modify. Indeed, self-modification was not obvious in the PUP-IT2 system (Figures 3B,D), indicating that PUP-IT2 has less labeling background from self-labeling.

PUP-IT2 is also different from all other proximity labeling methods in the way that the ligation of POI is a single turnover reaction while the others allow multiple turnovers. Giving the POI is fused to one substrate, it can only be attached to one proximal protein. This may partly explain why the PUP-IT2-mediated modification intensity is not as strong as PUP-IT in Figures 3B,D. The cellular labeling efficiency of PUP-IT2 can be affected by various factors, including cellular concentrations of the enzyme and substrate, the stoichiometry between the labeling tag and the substrate, and the different geometries between PupE and the target proteins. However, the modifications by PUP-IT2 are more evenly distributed with different molecular weights, representing diverse proximal proteins. By contrast, PUP-IT-mediated modifications are mainly above the molecular weight of the PafA fusions, suggesting dominated self-modifications. More starting materials for protein enrichment and mass spectrometry identification can overcome the disadvantage of single turnover by PUP-IT2.

In summary, PUP-IT2 is another proximity labeling strategy with the smallest fusion protein, almost no background self-labeling, and all genetically-encoded components for potential *in vivo* expression. It is expected to be applied in different contexts to study diverse biological mechanisms in a wide range of organisms.

## Methods

### Molecular cloning

Plasmids were constructed for either bacteria expression or mammalian cell expression. For recombinant protein expression in *E. coli*, *C. glutamicum* PafA (cg1688) or human SPOP MATH domain (28–166) was subcloned into pGEX6p-1 *BamH1* restriction enzyme cleavage site. Overlapping PCRs were used to generate His-SUMO-Myc-PEX3 (49-373)-PupE and His-SUMO-BCCP-pep1-PupE. A synthetic peptide was used as the template for His-SUMO. Human PEX3 was amplified from the cDNA library, BCCP and codon-optimized *C. glutamicum* Pup (cg1689) were cloned from Addgene constructs #113403 (Liu et al., 2018). The fusions were subcloned into the first multi-cloning site of pRSFDuet *BamH1* restriction enzyme cleavage site.

For protein expression in mammalian cells, Flag-PEX19-PupE, BCCP-PupE, BCCP-PEX19-PupE, Flag-PafA, NUP43-PafA-Flag, NUP43-BCCP-PupE, PEX3-Flag-PafA, and PEX3-BCCP-PupE were cloned into pCDNA3.1, using Gibson assembling. NUP43 was cloned from human genome cDNA.

### Recombinant protein expression and purification

Purification of His-tagged protein Plasmid (pRSFDuet) encoding His fused protein was transformed into *E. coli* BL21 (DE3). Cells were grown in 1 L of LB media supplemented with 50 μg/ml kanamycin at 37 °C until the OD_600_ reached 0.6. Protein expression was induced with 0.2 mM IPTG and cells continued to grow at 18 °C overnight. The cell culture was then harvested by centrifugation at 4500 × g for 15 min, resuspended in lysis buffer (50 mM Tris, pH 8.0, 200 mM NaCl) and lysed by a French pressure cell press. The supernatant was isolated by centrifugation for 1 h at 4°C at 48,000 × g and incubated with High Affinity Ni-Charged Resin (GenScript; L00666-5) on a gravity column. Ni-NTA resin was washed with 15 column volumes of wash buffer (50 mM Tris, pH 8.0, 200 mM NaCl, 20 mM imidazole) before being eluted with 5 ml column volumes of elution buffer (50 mM Tris, pH 8.0, 200 mM NaCl, 250 mM imidazole). The protein samples were aliquoted and stored at −80°C.

Purification of GST-tagged protein. Plasmid (pGEX6p-1) encoding GST fused protein was transformed into *E. coli* BL21(DE3). Cells were grown in 1 L of LB media supplemented with 100 μg/ml ampicillin at 37°C until the OD_600_ reached 0.6. Protein expression was induced with 0.2 mM IPTG and cells continued to grow at 18°C overnight. Cells were collected by centrifugation, resuspended in lysis buffer (50 mM Tris, pH 8.0, 200 mM NaCl, 1 M DTT), and lysed by a French pressure cell press. The supernatant was isolated by centrifugation for 1 h at 4°C at 48,000 × g and purified by *ProteinIso* ^®^ GST Resin (TransGen; DP201-01) on a gravity column. Glutathione resin was washed with 15 column volumes of lysis buffer before being eluted with 5 ml column volumes of elution buffer (50 mM Tris, pH 8.0, 200 mM NaCl, 1 M DTT, 10 mM reduced glutathione). For GST-PafA, after exchange the elution buffer with lysis buffer, precision protease was added at a ratio of 1:200 (w/w) and incubated at 4 °C overnight, then recombine with GST Resin to eliminate the GST.

### *In vitro* PUP-IT2 labeling assay

*In vitro* pupylation reactions were performed with 10 µM PUP-IT2, 10 µM labeling target protein, and 1 µM PafA in the reaction buffer containing 20 mM Tris, pH 8.0, 100 mM NaCl, 10 mM ATP, and 15 mM MgCl_2_. Reactions were prepared in 20 µL and assayed at 37°C for 30 min, then stopped by direct addition of 6× SDS loading buffer. In the PUP-IT2 peptide competition experiment, the synthetic free peptide pep1 (LACDEVTSTTSSSTA) and the PEX19 peptide (ADRELEELLESALDDFDKAK) interacting with PEX3 were synthesized and purified to >98% (Changzhou Kanglong Biotech Ltd.), the synthesized peptides were dissolved in water and adjusted to pH 7 with Tris, pH 8.0. All the reactions were stopped by direct addition of 6× SDS loading buffer and analyzed on 4–20% SDS-PAGE gels (GenScript, M42012C), then subjected to Coomassie brilliant blue staining and immunoblotting with anti-GST (Cell Signaling, 2622S), anti-His (Cell Signaling, 9991S), anti-PEX3 (ABclonal, A7352) and anti-PEX19 (Proteintech, 14713-1-AP) to identify the conjugated bands.

### Cell culture and transient transfection

HEK293 cells (ATCC, CRL-1573) and HeLa cells (ATCC, CCL-2) were cultured in DMEM (Thermo; C11995500CP) supplemented with 10% FBS (GEMINI; 900-108) in 5% CO_2_ at 37°C. Transient transfections were performed in HeLa cells using the Lipofectamine 3000 reagent (Thermo, L3000015) according to the manufacturer’s instructions. HEK293 cells were transfected with a mix of plasmid and PEI (polyethyleneimine, 9002-98-6) at a weight ratio of 1:2 using PEI transfection protocol (Yang et al., 2017). All the cell lines were also tested and confirmed negative for *mycoplasma*.

### Cellular PUP-IT2 labeling assay

For PupE labeling of transiently transfected cells, two plasmids were co-transfected into wild-type HEK293 cells, one containing PUP-IT2 fusion protein such as PEX3-PupE and the other containing PafA. Exogenous biotin (0.4 M stock in DMSO) was diluted in complete media and added directly to cells to a final concentration of 4 μM after transfection for 24 h. The expression of fusion proteins and the labeling of PUP-IT2 system were verified by immunoblotting.

### Immunoblot and immunofluorescence

Proteins were separated on 4–20% SDS-PAGE gels and transferred to polyvinylidene fluoride membranes (Millipore, SLGVR33RS). After blocking with 5% non-fat powdered milk (BBI, A600669-0250) in TBST (20 mM Tris, pH 7.5, 1% Tween-20, 150 mM NaCl) for 1 h, membranes were incubated with primary antibody or horseradish peroxidase (HRP)-conjugated streptavidin at room temperature for 1 h. The primary antibodies were detected using HRP-conjugated secondary antibodies and signals from antibodies were detected using enhanced chemiluminescence (EpiZyme, SQ201) for immunofluorescence imaging.

Cells were rinsed three times with PBS with gentle shaking and fixed in 4% PFA (Paraformaldehyde) for 15 min, followed by PBS washing for three times. After 10 min of membrane rupture (0.1% NP40 in PBS) and 1 h of blocking (2% BSA in Cell Staining Buffer, 4A Biotech, FXP005), cells were stained with the following primary antibodies: anti-Flag (GNI, GNI4110-FG), anti-PMP70 (ABclonal, A4172). Alexa Fluor 488 conjugated anti-mouse and Alexa Fluor-555 conjugated anti-rabbit antibodies were used for secondary staining, and samples were mounted using Mounting Medium (Sigma; DUO82040). Confocal fluorescence imaging was performed using Zeiss LSM 800 confocal microscope with a 63× oil plan APO.

### Mass spectrometry sample preparation and analysis

For each cell sample in mass spectrometry analysis, PUP-IT2 transfected wild type HEK293 cells were grown to 1 × 10^8^ cells then harvested and lysed by 10 ml lysis buffer (50 mM Tris, 200 mM NaCl, 1% NP-40, pH 7.5) supplemented with protease inhibitor (APExBIO, K1007). Then, urea powder was added to cell lysate to the final concentration at 8 M, Cysteine carbamidomethylate labeling was performed with sequential 10 mM DTT at 56°C (1 h), 25 mM iodoacetamide treatment in the dark for 45 min, and quench with additional 25 mM DTT. 400 µL neutravidin agarose resin (Thermo, 29200) were added into the lysate and incubated on a rotator at room temperature for 1 h. Beads were washed extensively with buffer 1 (50 mM Tris, pH 8.0, 8 M urea, 200 mM NaCl, 0.2% SDS), buffer 2 (50 mM Tris, pH 8.0, 8 M urea, 200 mM NaCl, 2% SDS), buffer 3 (50 mM Tris, pH 8.0, 8 M urea, 200 mM NaCl) and buffer 4 (50 mM Tris, pH 8.0, 0.5 mM EDTA, 1 mM DTT) sequentially. Finally, beads were resuspended with 200 µL 100 mM ammonium carboxylate, and 30 μL trypsin (Promega, V5113) was added for on-bead digestion over-night at 37°C. The digested peptides were collected and cleaned with SOLAµ™ plates (ThermoFisher; 60209-001) before MS analysis.

Liquid Chromatography−Mass Spectrometry Reversed-phase capillary HPLC separations were performed using an EASY-nLC 1200 UPLC system coupled in-line with a Thermo Orbitrap Fusion tribrid mass spectrometer (Thermo Scientific, Bremen, Germany). 3 μL peptides (equivalent to 1 μg) were loaded and resolved on an analytical column. The gradient was comprised of an increase from 6% to 34% solvent B (0.1% FA in 80% ACN) over 50 min, 34%–38% in 3 min and climbing to 90% in 1 min then holding at 90% for the last 6 min, all at a constant flow rate of 250 nL/min on an EASY-nLC 1200 UPLC system. The resulting peptides were analyzed by Orbitrap Fusion mass spectrometer (ThermoFisher Scientific). MS(/MS) data were acquired on an Orbitrap Fusion as follows: All MS1 spectra were acquired over m/z 350–1400 in the orbitrap (120 K resolution at 200 m/z); automatic gain control (AGC) was set to accumulate 5 × 105 ions, with a maximum injection time of 50 ms. Data-dependent tandem MS analysis was performed using a top-speed approach (cycle time of 3 s). The normalized collision energy was optimized at 28% for HCD. MS2 spectra were acquired with a fixed first m/z of 100. The intensity threshold for fragmentation was set to 50 000 for orbitrap methods and included charge states 2 + to 6+. A dynamic exclusion of 30 s was applied with a mass tolerance of 10 ppm. AGC was set to 50,000 with a maximum injection time set at 50 ms for OT; Data Analysis Data were processed using Andromeda integrated in MaxQuant (1.6.5.0) using default settings unless otherwise specified. Tandem mass spectra were searched against human database. Trypsin/P was specified as cleavage enzyme allowing up to 2 missing cleavages. Mass error was set to 10 ppm for precursor ions and 0.02 Da for fragment ions. Carbamidomethyl on Cys were specified as fixed modification and oxidation on Met was specified as variable modifications. Peptide ion score was set >20.

The mass spectrometry proteomics data have been deposited to the ProteomeXchange Consortium (http://proteomecentral.proteomexchange.org) *via* the iProX partner repository (Ma et al., 2019) with the dataset identifier PXD036047.

### Statistics

The raw data were processed and searched with MaxQuant 1.6.5.0. Venn diagram were draw using Biovenn website (http://www.biovenn.nl). Heatmap were drawn using the hiplot website (https://hiplot-academic.com) based on the MS counts. Sub-cellular location and protein-association networks was analysis by STRING website (https://cn.string-db.org).

## Data Availability

The datasets presented in this study can be found in online repositories. The names of the repository/repositories and accession number(s) can be found below: http://proteomecentral.proteomexchange.org/cgi/GetDataset PXD036047.

## Supplementary material

Supplementary Figure S1 PUP-IT2 has less non-specific labeling. *In vitro* Pup modification assay of PUP-IT2^PEX3^. 10 µM His-PEX19 or BSA were used for the reaction. Proteins were analyzed with SDS-PAGE for Coomassie stain and western blotting, using anti-PEX3 and anti-PEX19 antibodies.
