# Supplementary material for: PUP-IT2 as an alternative strategy for PUP-IT proximity labeling
Source: Front Mol Biosci. 2022 Sep 29;9:1007720. doi: 10.3389/fmolb.2022.1007720 (PMC9558124; doi:10.3389/fmolb.2022.1007720)
Supplement: Supplementary file 2 [file Image-1.pdf]

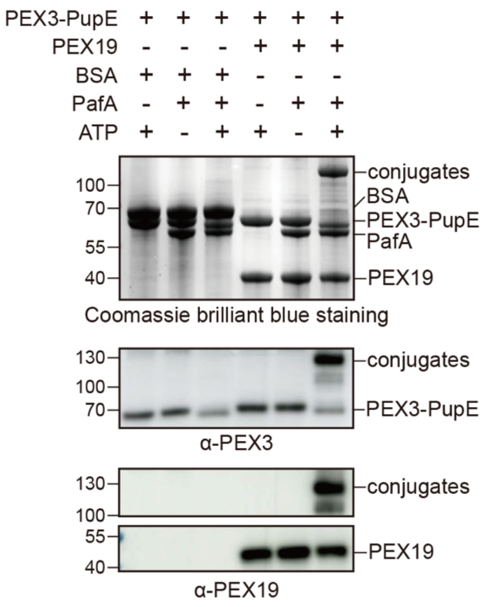

Fig. S1 PUP-IT2 has less non-specific labeling. *In vitro* Pup modification assay of PUP-IT2^{PEX3}. 10 μM His-PEX19 or BSA were used for the reaction. Proteins were analyzed with SDS-PAGE for Coomassie stain and western blotting, using anti-PEX3 and anti-PEX19 antibodies.
